# A Peculiar Case of Irritation of the Nervous System, in a Child Whilst Teething

**Published:** 1878-02

**Authors:** A. Blumberg

**Affiliations:** Pittsburg, Pa.


### A PECULIAR CASE OF IRRITATION OF THE NERVOUS SYSTEM, IN A CHILD, WHILST TEETHING.

BY DR. A. BLUMBERG, PITTSBURG, PA.

*Read before the Allegheny Medical Society, at their last meeting, September 18, 1877.*

On the 8th of last August I was called to see Latie R., aged ten months, living on Thumm street, Fourteenth Ward. Two days previously she was taken sick with choleraic diarrhœa, which attack recurred at intervals. I found the child very restless and irritable; pulse small and rapid, but no fever. Upon examination of the gums, found the upper one red, swollen and very sensitive to the touch. I prescribed the following:

| | | | |
|---|---|---|---|
| ℞ | Mag. sulph., | ℨj | |
| | Tinct. rhei., | ℨij | |
| | Syr. zingiber., | ℨj | |
| | Aqua menth., | ℨix | M. |

Sig. A teaspoonful every three hours.

On the same evening, at ten o'clock, was sent for in great haste, the messenger stating that the child, who during the day had appeared better, had suddenly become cold all over, and was lying unconscious. On my arrival at the house (which was about thirty minutes after the child had been taken with this attack), I found her apparently unconscious; the body rather cool; face, head and especially the ears very cold, and rather moist; the temperature in the axilla was normal, the pulse full and regular, about seventy. Upon calling her by name she gave no sign of recognition, but stirred slightly when pinched. Patient appeared as if overcome by deep sleep from which she was powerless to awaken. Gave her brandy and hot tea, which she swallowed but remained in this deep sleep until eleven o'clock; she then commenced slowly to awaken, the coolness of the skin gradually becoming lessened, until it reached its normal temperature, when she showed a desire for nourishment, and seemed to be rather prostrated.

August 9th, a.m. Patient improving; vomiting entirely stopped; diarrhœa reduced to five or six passages in the last twelve hours; temperature 101°. Medicine to be continued. 9:30 p.m. Had another attack, of the same intensity and duration as on the previous evening.

August 10th, a. m. Temperature 101.2°; bowels move less frequently and of fecal consistence. Ordered sulphate of quinia two grain doses. every two hours, commencing four hours before the attack comes on. 8:30 p.m. took another attack which lasted until 10 p.m.

August 11th, 10 a.m. Had no passage in the last eight hours; temperature 101°. Ordered the quinine to be given in four grain doses an hour before the attack, which, however, came on at eight o'clock and lasted until midnight.

August 12th, a. m. Bowels still confined. Ordered an enema of tepid water and castor oil; discontinued the quinine, and prescribed oxide of zinc, in half grain doses, every three hours.

At 7:30 p.m. Another attack, which lasted until 11 p. m.

August 13th, a. m. The child laughing and playing; bowels still confined. Medicine to be continued, and a teaspoonful of castor oil to be given.

7 p. m. The attack came on whilst the child was playing in her mother's arms, lasting until 9 p. m.

These attacks recurred on the evenings of the 14th and 15th of August, but were of shorter duration and of less intensity

August 16th, a. m. The child seems to be in usual health; bowels moved once this morning; temperature 98°. The mother, who at my request had examined the child's mouth daily during her sickness, stated that, to her great joy, she had found. this morning, the first appearance of the third upper molar. She had no return of the attack that evening, nor has she had any since, and is in perfect health.

The question now arises, to what disorder can these attacks be attributed?

That these periodical spells were connected with dentition at that time is proved, first by the febrile symptoms present during the day, from the 9th until the 16th. Second, by the disordered condition of the bowels, which generally accompanies teething. Third, the cessation of all these symptoms, on the morning of the 16th, after the molar tooth had made its appearance. Taking this into consideration, and the form in which these attacks occurred, we are compelled to look for the cause in some disorder of the nervous system. brought on either by direct irritation of the centre of the nervous system, or by reflex irritation of some other organ.

The query now is, in what district of the nervous system must we look for the cause of such disturbance?

We know from physiology that the blood vessels are kept in a steady tension (called tonus) by influence of the nervous system, which, according to an experiment by Schiff, was proved to be the function of the nerve sympatheticus. Schiff found, by experimenting on rabbits, that by cutting through the sympathetic nerve on the neck, the corresponding side of the head, face, and more particularly the ears, became flushed and hot, the arteries becoming thereby dilated.

By faradaical irritation of the same nerve, the corresponding side of the head, face, and especially the ears become cold, the arteries becoming contracted.

I believe therefore, that in this case of Katie R., the remittent attacks were caused by some irritation of the sympathetic nerve, brought on either by the irritable condition of the brain during the period of teething, or by reflex irritatian of some other disordered organ at that time. I have never read of a similar case; and, therefore, deem this sufficient interest to the profession to bring it to your notice.—*Med. and Surg. Reporter.*

